# Hepatic Sarcodosis presenting as portal hypertension in a young boy 

**Published:** 2018

**Authors:** Inamullah Khan Achakzai, Zain Majid, Muhammad Ali Khalid, Shoaib Ahmed Khan, Syed Mudassir Laeeq, Nasir Hassan Luck

**Affiliations:** *Department of Hepato-gastroenterology, Sindh Institute of Urology and Transplantation, (SIUT), Civil Hospital Karachi 74200, Karachi, Pakistan *

**Keywords:** Hepatic sarcoidosis, chronic granulomatous disease, steroids, Serum ACE levels

## Abstract

A 13-year-old boy, known case renal stone disease came with the complaints of abdominal pain along with low grade fever. On examination, hepatosplenomegaly was noted while his lab reports showed a low hemoglobulin with a raised ESR. His blood and urine cultures showed no growth. Viral markers, autoimmune profile, C and p ANCA were all negative apart from a raised serum IgG level. Ultrasound abdomen showed a hyperechoic liver with an enlarged spleen along with splenic varices and minimum ascites. Ultrasound hepatic doppler was normal. Serum AFP levels were normal while workup for Wilson’s disease was negative. Fibroscan showed F4 fibosis. CT scan abdomen showed an enlarged left lobe of the liver along with an enlarged spleen. His EGD revealed varices. So liver biopsy was done that was suggestive of chronic granulomatous disease with ZN stain testing negative for TB.PPD, urine for AFB were both negative. Serum ACE levels were raised. He started ATT therapy but his condition did not improve. So, on the suspicion of hepatic sarcoidosis, he started on steroids and had a drastic improvement in his condition.

## Introduction

Sarcoidosis is a disease of unknown etiology that can affect the various organs of the body. It is seen most often in females and has a prevalence of 2-60 cases per 100,000 people around the world (1).

## Case report

A young boy, having a history of bilateral renal stone disease, presented to our outpatients’ department having generalized abdominal pain along with low grade fever. On examination he appeared pale and his liver was palpable 2 finger breadths below the right costal margin while his spleen was also palpable. His initial laboratory works showed a Hemoglobin (Hb) 8.3 g/dl, MCV 90.3 fL/red cell, total leukocyte count (TLC) 4900, platelet count of 190k, PT 12.1, APTT 20, INR 1.15, while his serum creatinine was 1.2mg/dL. His liver function tests (LFTs) were normal while his ESR was raised (48 mm/hr). His chest x-ray (CXR) was normal. Based upon his previous history of renal stone disease, a urine detailed report (D/R) was ordered, which unremarkable and blood culture and sensitivity showed no growth (which were done to rule out any infective cause).

Viral markers were also sent and were negative. His autoimmune profile (ANA, AMA, ASMA, anti LKM) were all negative. Perinuclear Anti-Neutrophil Cytoplasmic Antibodies (P ANCA) and Cytoplasmic antineutrophil cytoplasmic antibodies (c ANCA) were also negative. While serum immunoglobulin G (IgG) levels were raised (21).

His ultrasound abdomen showed a liver of 16.6 cm, which was hyperechoic, having irregular margins. No space occupying lesions noted in it. Portal vein was of 1.4 cm in diameter, while his spleen was of 20.6 cm having splenic varices. Minimal ascities was also noted. Ultrasound hepatic doppler showed all hepatic vessels and portal vein to be patent.

We then requested for serum alpha feto protein (AFP) levels, which was within normal limits. Serum ceruloplasmin levels and 24-hour urinary copper levels were also within the normal range while no evidence of Kayser-Fleischer Ring (KF ring) was noted on ophthalmological examination.

A fibroscan of the liver and F4 Fibrosis were noted. Later on, a CT scan abdomen was planned and it showed hepatosplenomegaly along with few splenic varices. Few small mediastinal lymph nodes were also seen in superior mediastinum.

Upper gastrointestinal endoscopy was done and it revealed one column of grade III and 2 columns of grade II esophageal varices with mild portal gastropathy.

**Figure 1:**
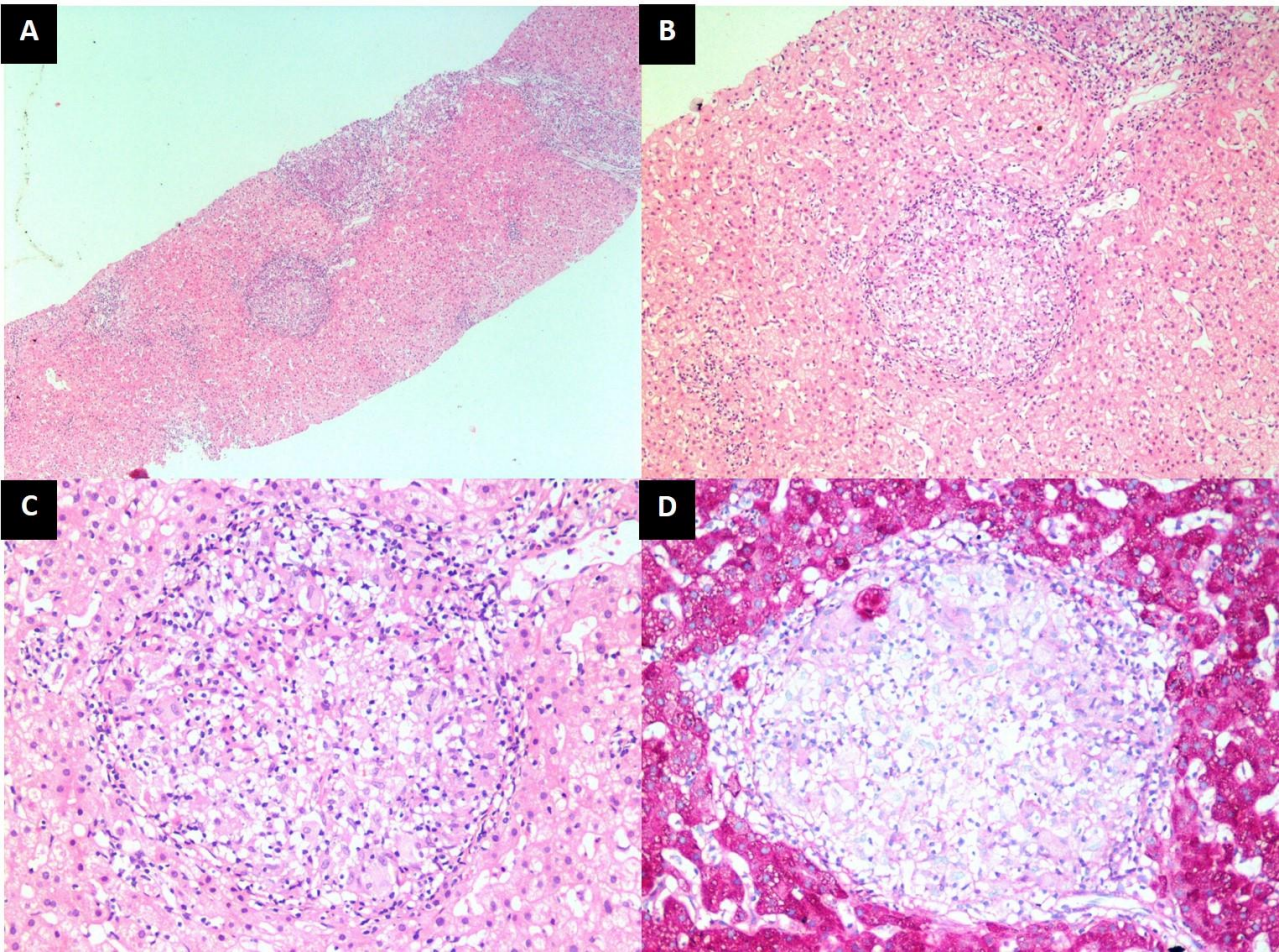
Histopathological features on liver biopsy. A. Low-power view showing portal and lobular inflammation. (H&E, ×40). B. Medium-power view a well-formed epithelioid granuloma in the lobular parenchyma surrounded by sparse lymphocytic infiltrate (H&E, ×100). C. High-power view showing collection of epithelioid cells with a few giant cells and lymphocytes. (H&E, ×200). D. Same granuloma highlighted by PAS stain. (PAS, ×200

To further aid in our diagnostic effort, we proceeded with a liver biopsy, which showed partial effacement of lobular architecture. Portal tract was moderately expanded with fibrosis and mixed inflammatory cells infiltrate with predominant lymphocytes and few eosinophils. There were many well formed epitheloid granulomata in the portal tract and as well as in the liver parenchyma. No caseation necrosis was seen. Florid lobulitis and piecemeal necrosis were noted. No portal top portal tract bridging fibrosis were noted. No siderosis, steatosis or cholestasis were seen. Ziehl Neelson (ZN stain) was negative. The morphological features were suggestive of chronic granulomatous disease. 

 Tuberculin skin test (PPD) was injected and the induration was found to be of less than 5mm.3 samples of urine for acid fast bacilli (AFB Smear) were also negative. Serum angiotensin converting enzyme (ACE) levels were raised.

He was initially started on Anti tuberculosis therapy (ATT) on the suspicion of tuberculosis however after two months it was stopped on account of lack of improvement of his symptoms and negative tests for Tuberculosis. On the suspicion of hepatic sarcoidosis, he was started on steroids and marked improvement in his condition was seen. 

## Discussion

Sarcoidosis is a disease that has multi-system involvement along with the presence of non-caseating granuloma in the involved organ (1). Pulmonary involvement is mainly seen but it can also have hepatic involvement 1. Hepatic sarcoidosis was seen in 11.5% of the patients enrolled in the Access study, which was a case control etiologic study of sarcoidosis (2).

 Patients having hepatic sarcoidosis are mostly asymptomatic and their lab works can show deranged LFTs (1). Diagnosing hepatic sarcoidosis is a difficult task since no definite diagnostic test or lab investigation is currently available for it.3 Patients with hepatic sarcoidosis usually complain of abdominal pain, localized to the right hypochondrium along with jaundice and pruritis (1). Cirrhosis and portal hypertension are long term complications of hepatic sarcoidosis (1).

Tan CB *et al.* showed hepatic sarcoidosis with portal hypertension along with cirrhosis of liver, which was a similar presentation to the one seen in our case (3).

Since hepatic sarcoidosis needs to be differentiated from autoimmune diseases and other granulomatous diseases, its diagnosis relies on the liver biopsy findings (1). Before treatment starts, one must exclude other granulomatous diseases (4).

Corticosteroids are the main treatment options and liver transplant is the definite treatment of choice (4). Another study showed the role of ursodeoxycholic acid (UDCA) used empirically for hepatic sarcoidosis (5).

It is also important to differentiate hepatic sarcoidosis from Tuberculosis (Tb) or lymphoma, since the typical lab workup for sarcoidosis is not found to be helpful in establishing a diagnosis (6).

One case series showed that although steroids are the mainstay of therapy for hepatic sarcoid, antimalarials and UDCA in combination were helpful in treatment of steroid resistant sarcoidosis (7).

Our case reflects the fact that in a Tuberculosis (Tb) endemic country like ours, sarcoidosis can still occur and should only be suspected after other causes have been ruled out.

## Conflict of interests

The authors declare that they have no conflict of interest.
